# The bright side of sports: a systematic review on well-being, positive emotions and performance

**DOI:** 10.1186/s40359-024-01769-8

**Published:** 2024-05-21

**Authors:** David Peris-Delcampo, Antonio Núñez, Paula Ortiz-Marholz, Aurelio Olmedilla, Enrique Cantón, Javier Ponseti, Alejandro Garcia-Mas

**Affiliations:** 1 General Psychology Department, Valencia University, Valencia, 46010 Spain; 2 Basic Psychology and Pedagogy Departments, Balearic Islands University, Palma de Mallorca, 07122 Spain; 3 Education and Social Sciences Faculty, Andres Bello University, Santiago, 7550000 Chile; 4 Personality, Evaluation and Psychological Treatment Deparment, Murcia University, Campus MareNostrum, Murcia, 30100 Spain

**Keywords:** Well-being, Positive emotions, Sports performance

## Abstract

**Objective:**

The objective of this study is to conduct a systematic review regarding the relationship between positive psychological factors, such as psychological well-being and pleasant emotions, and sports performance.

**Method:**

This study, carried out through a systematic review using PRISMA guidelines considering the Web of Science, PsycINFO, PubMed and SPORT Discus databases, seeks to highlight the relationship between other more ‘positive’ factors, such as well-being, positive emotions and sports performance.

**Settings:**

The keywords will be decided by a Delphi Method in two rounds with sport psychology experts.

**Participants:**

There are no participants in the present research.

**Assessment:**

The main exclusion criteria were: Non-sport thema, sample younger or older than 20–65 years old, qualitative or other methodology studies, COVID-related, journals not exclusively about Psychology.

**Main outcomes measures:**

We obtained a first sample of 238 papers, and finally, this sample was reduced to the final sample of 11 papers.

**Results:**

The results obtained are intended to be a representation of the ‘bright side’ of sports practice, and as a complement or mediator of the negative variables that have an impact on athletes’ and coaches’ performance.

**Conclusions:**

Clear recognition that acting on intrinsic motivation continues to be the best and most effective way to motivate oneself to obtain the highest levels of performance, a good perception of competence and a source of personal satisfaction.

## Introduction

In recent decades, research in the psychology of sport and physical exercise has focused on the analysis of psychological variables that could have a disturbing, unfavourable or detrimental role, including emotions that are considered ‘negative’, such as anxiety/stress, sadness or anger, concentrating on their unfavourable relationship with sports performance [1–4], sports injuries [5–7] or, more generally, damage to the athlete’s health [8–10]. The study of ‘positive’ emotions such as happiness or, more broadly, psychological well-being, has been postponed at this time, although in recent years this has seen an increase that reveals a field of study of great interest to researchers and professionals [11–13] including physiological, psychological, moral and social beneficial effects of the physical activity in comic book heroes such as Tintin, a team leader, which can serve as a model for promoting healthy lifestyles, or seeking ‘eternal youth’ [14].

Emotions in relation to their effects on sports practice and performance rarely go in one direction, being either negative or positive—generally positive and negative emotions do not act alone [15]. Athletes experience different emotions simultaneously, even if they are in opposition and especially if they are of mild or moderate intensity [16]. The athlete can feel satisfied and happy and at the same time perceive a high level of stress or anxiety before a specific test or competition. Some studies [17] have shown how sports participation and the perceived value of elite sports positively affect the subjective well-being of the athlete. This also seems to be the case in non-elite sports practice. The review by Mansfield et al. [18] showed that the published literature suggests that practising sports and dance, in a group or supported by peers, can improve the subjective well-being of the participants, and also identifies negative feelings towards competence and ability, although the quantity and quality of the evidence published is low, requiring better designed studies. All these investigations are also supported by the development of the concept of eudaimonic well-being [19], which is linked to the development of intrinsic motivation, not only in its aspect of enjoyment but also in its relationship with the perception of competition and overcoming and achieving goals, even if this is accompanied by other unpleasant hedonic emotions or even physical discomfort. Shortly after a person has practised sports, he will remember those feelings of exhaustion and possibly stiffness, linked to feelings of satisfaction and even enjoyment.

Furthermore, the mediating role of parents, coaches and other psychosocial agents can be significant. In this sense, Lemelin et al. [20], with the aim of investigating the role of autonomy support from parents and coaches in the prediction of well-being and performance of athletes, found that autonomy support from parents and coaches has positive relationships with the well-being of the athlete, but that only coach autonomy support is associated with sports performance. This research suggests that parents and coaches play important but distinct roles in athlete well-being and that coach autonomy support could help athletes achieve high levels of performance.

On the other hand, an analysis of emotions in the sociocultural environment in which they arise and gain meaning is always interesting, both from an individual perspective and from a sports team perspective. Adler et al. [21] in a study with military teams showed that teams with a strong emotional culture of optimism were better positioned to recover from poor performance, suggesting that organisations that promote an optimistic culture develop more resilient teams. Pekrun et al. [22] observed with mathematics students that individual success boosts emotional well-being, while placing people in high-performance groups can undermine it, which is of great interest in investigating the effectiveness and adjustment of the individual in sports teams.

There is still little scientific literature in the field of positive emotions and their relationship with sports practice and athlete performance, although their approach has long had its clear supporters [23, 24]. It is comforting to observe the significant increase in studies in this field, since some authors (e.g [25, 26]). . , point out the need to overcome certain methodological and conceptual problems, paying special attention to the development of specific instruments for the evaluation of well-being in the sports field and evaluation methodologies.

As McCarthy [15] indicates, positive emotions (hedonically pleasant) can be the catalysts for excellence in sport and deserve a space in our research and in professional intervention to raise the level of athletes’ performance. From a holistic perspective, positive emotions are permanently linked to psychological well-being and research in this field is necessary: firstly because of the leading role they play in human behaviour, cognition and affection, and secondly, because after a few years of international uncertainty due to the COVID-19 pandemic and wars, it seems ‘healthy and intelligent’ to encourage positive emotions for our athletes. An additional reason is that they are known to improve motivational processes, reducing abandonment and negative emotional costs [11]. In this vein, concepts such as emotional intelligence make sense and can help to identify and properly manage emotions in the sports field and determine their relationship with performance [27] that facilitates the inclusion of emotional training programmes based on the ‘bright side’ of sports practice [28].

Based on all of the above, one might wonder how these positive emotions are related to a given event and what role each one of them plays in the athlete’s performance. Do they directly affect performance, or do they affect other psychological variables such as concentration, motivation and self-efficacy? Do they favour the availability and competent performance of the athlete in a competition? How can they be regulated, controlled for their own benefit? How can other psychosocial agents, such as parents or coaches, help to increase the well-being of their athletes?

This work aims to enhance the leading role, not the secondary, of the ‘good and pleasant side’ of sports practice, either with its own entity, or as a complement or mediator of the negative variables that have an impact on the performance of athletes and coaches. Therefore, the objective of this study is to conduct a systematic review regarding the relationship between positive psychological factors, such as psychological well-being and pleasant emotions, and sports performance. For this, the methodological criteria that constitute the systematic review procedure will be followed.

## Materials and methods

This study was carried out through a systematic review using PRISMA (Preferred Reporting Items for Systematic Reviews) guidelines considering the Web of Science (WoS) and Psycinfo databases. These two databases were selected using the Delphi method [29]. It does not include a meta-analysis because there is great data dispersion due to the different methodologies used [30].

The keywords will be decided by the Delphi Method in two rounds with sport psychology experts. The results obtained are intended to be a representation of the ‘bright side’ of sports practice, and as a complement or mediator of the negative variables that have an impact on athletes’ and coaches’ performance.

It was determined that the main construct was to be psychological well-being, and that it was to be paired with optimism, healthy practice, realisation, positive mood, and performance and sport. The search period was limited to papers published between 2000 and 2023, and the final list of papers was obtained on February 13^,^ 2023. This research was conducted in two languages—English and Spanish—and was limited to psychological journals and specifically those articles where the sample was formed by athletes.

Each word was searched for in each database, followed by searches involving combinations of the same in pairs and then in trios. In relation to the results obtained, it was decided that the best approach was to group the words connected to positive psychology on the one hand, and on the other, those related to self-realisation/performance/health. In this way, it used parentheses to group words (psychological well-being; or optimism; or positive mood) with the Boolean ‘or’ between them (all three refer to positive psychology); and on the other hand, it grouped those related to performance/health/realisation (realisation; or healthy practice or performance), separating both sets of parentheses by the Boolean ‘and’’. To further filter the search, a keyword included in the title and in the inclusion criteria was added, which was ‘sport’ with the Boolean ‘and’’. In this way, the search achieved results that combined at least one of the three positive psychology terms and one of the other three.

## Results

### Results (first phase)

**Fig. 1:**
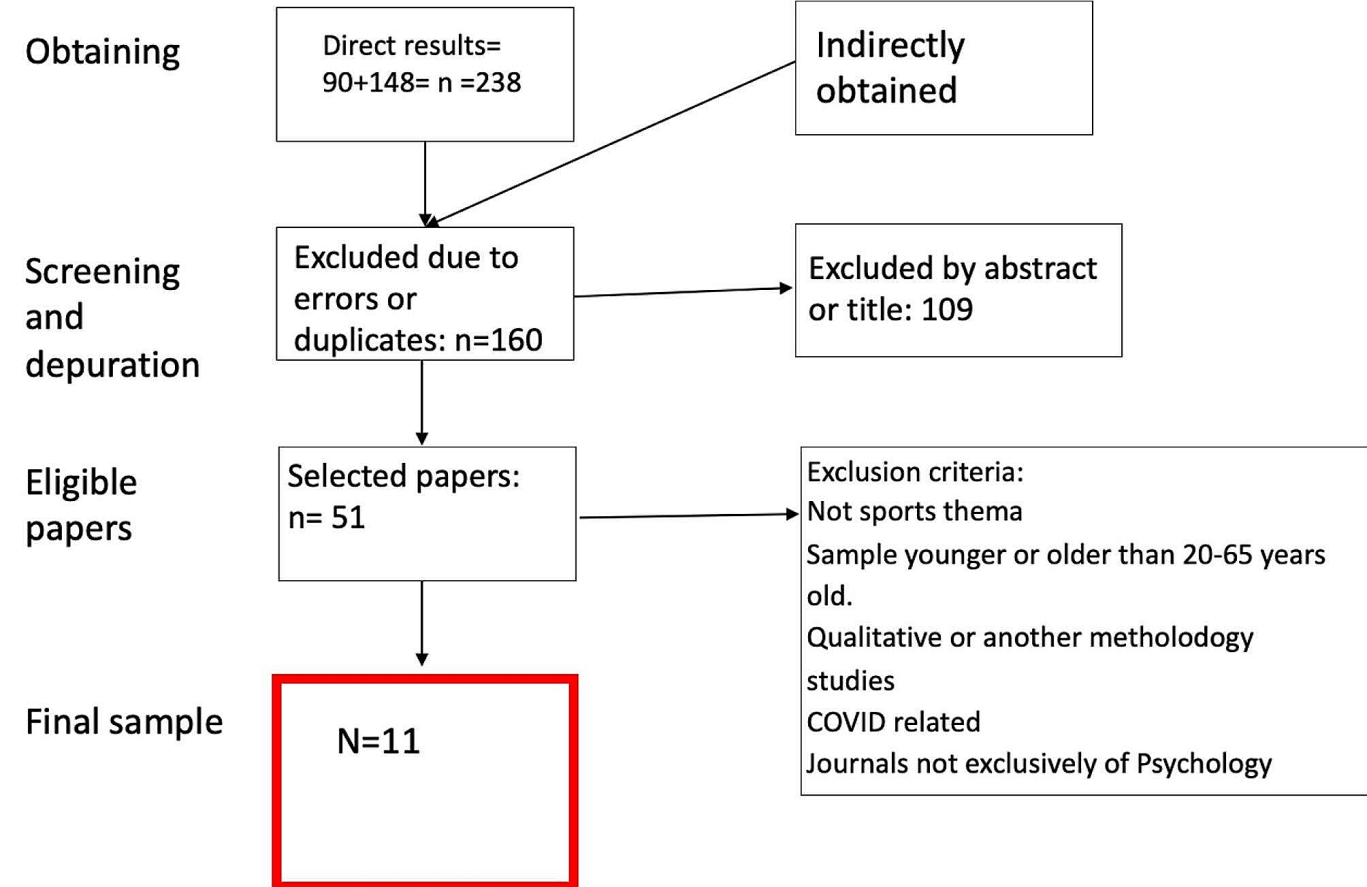
Phases of the selection process for the final sample. Four phases were carried out to select the final sample of articles. The first phase allowed the elimination of duplicates. In the second stage, those that, by title or abstract, did not fit the objectives of the article were eliminated. Previously selected exclusion criteria were applied to the remaining sample. Thus, in phase 4, the final sample of 11 selected articles was obtained

The mentioned keywords were cross-matched, obtaining the combination with a sufficient number of papers. From the first research phase, the total number of papers obtained was 238. Then screening was carried out by 4 well-differentiated phases that are summarised in Fig. 1. These phases helped to reduce the original sample to a more accurate one.

### Results (second phase)

The first screening examined the title, and the abstract if needed, excluding the papers that were duplicated, contained errors or someone with formal problems, low N or case studies. This screening allowed the initial sample to be reduced to a more accurate one with 109 papers selected.

### Results (third phase)

This was followed by the second screening to examine the abstract and full texts, excluding if necessary papers related to non-sports themes, samples that were too old or too young for our interests, papers using qualitative methodologies, articles related to the COVID period, or others published in non-psychological journals. Furthermore, papers related to ‘negative psychological variables’’ were also excluded.

### Results (fourth phase)

At the end of this second screening the remaining number of papers was 11. In this final phase we tried to organise the main characteristics and their main conclusions/results in a comprehensible list (Table 1). Moreover, in order to enrich our sample of papers, we decided to include some articles from other sources, mainly those presented in the introduction to sustain the conceptual framework of the concept ‘bright side’ of sports.

**Table 1:** Final sample of articles selected to analyse in this systematic review

Nº	Paper	Authors	Journal	Year	Sample	Measure	Results	Main conclusions
1	Approach-achievement goals and motivational context on psychophysiological functioning and performance among novice basketball players	Mairi Mulvennaa, James W. Adie, Luke D. Sage, Nigel E. Wilson, Douglas Howat	Psychology of Sport & Exercise	2020	114 participants (M age = 23.53; SD = 4.56)	**Physiological**: HR* and BP** **Psychological**: stress appraisals, state anxiety, task enjoyment, perceived competence, and goal attainment	ANOVAs revealed that those who performed in a control motivational context reported significantly higher HR, systolic, and diastolic BP after the task. Those who adopted a task-approach goal in control conditions evaluated the shooting task as significantly more threatening. Pursuing approach-based goals in an autonomy-supportive context significantly improved their performance.	Limited support for an integrated motivational model.
2	Exploring Self-Compassion and Versions of Masculinity in Men Athletes	Nathan A. Reis, Kent C. Kowalski, Amber D. Mosewich, and Leah J. Ferguson	Journal of Sport and Exercise Psychology	2019	172 men athletes (M age = 22.8 yrs.)	- **Self-Compassion Scale** (SCS; Neff, 2003a) **- Rosenberg Self-Esteem** **Scale** (Rosenberg, 1965) **- Psychological Well-Being** (SPWB; Ryff & Keyes, 1995), - **Attitudes Toward Gay Men and Lesbians revised subscale** (ATG-R-S5; Herek, 1997; Herek, 1984) **- Subscale of the Internalised Shame Scale** (ISS; del Rosario & White, 2006). **- Self-Stigma of Seeking Help Scale** (Vogel et al., 2006). **- Performance Failure Appraisal Inventory** (Conroy et al., 2002). **-Fear of Negative Evaluation Scale** (Leary, 1983). - Other variables measured (i.e., **rumination**—Puterman et al., 2010; **self-criticism**—Gilbert & Procter, 2006; **concern over mistakes**—Gotwals & Dunn, 2009), as adapted by Mosewich et al. (2013). - **Sport Multidimensional Perfectionism Scale-2**; Gotwals & Dunn, 2009). - **Fears of Compassion Scales** (Gilbert et al., 2011), - **Inclusive Masculinity Theory Scale** (IMT). - **Conformity to Masculine Norms Inventory** (CMNI-46; Parent & Moradi, 2009).	This research explores the interaction of self-compassion and diverse versions of masculinity on the psychosocial well-being of men athletes. Self-compassion was related to most variables (e.g., psychological well-being, fear of negative evaluation, stated self-criticism, internalised shame, reactions to a hypothetical sport-specific scenario) in hypothesised directions. In addition, self-compassion was differentially related to inclusive and hegemonic masculinity.	Self-compassion as a promising resource for men athletes to buffer emotionally difficult sport experiences.
3	Relationships between mental toughness and psychological wellbeing in undergraduate students	Stamp, Elizabeth; Crust, Lee; Swann, Christian; Perry, John; Clough, Peter; Marchant, David	Personality and Individual Differences	2015	168 students from nine United Kingdom universities	**-The SPWB** (Ryff, 1989) **-The MTQ48** (Clough et al., 2002)	Multiple linear regression analyses found that components of mental toughness (MT) were moderate to strong predictors of psychological well-being (PWB) with between 35% and 64% of variance explained. Importantly, age, gender, and level of study were not found to predict PWB. These findings demonstrate the potential importance of MT within educational settings.	The potential importance of mental toughness within educational settings.
4	Stress mindset in athletes: Investigating the relationships between beliefs, challenge and threat with psychological wellbeing	Paul C. Mansell	Psychology of Sport & Exercise	2021	415 athletes (227 females, M age = 33.86 years, SD = 17.73)	**- Stress Mindset Measure - General** (SMM-G; Crum et al., 2013). **- The irrational performance beliefs inventory** (iPBI; Turner et al., 2016) **- The Challenge and Threat in Sport Scale** (CAT-Sport Scale; Rossato et al., 2018) **- The Patient Health Questionnaire** (PHQ-9; Kroenke et al., 2001) . **-Subjective Vitality Scale** (SVS; Ryan & Frederick, 1997).	Findings demonstrate that the relationship between stress mindset, irrational beliefs and psychological well-being is linked to direct and indirect effects of challenge and threat in athletes. Stress mindset was positively associated with challenge and negatively associated with threat, whilst self-depreciation and awfulizing demonstrated significant positive associations with threat. Self-depreciation was found to be significantly related to stress mindset, challenge, threat and depressive symptoms to a greater extent than the other types of irrational beliefs.	The stress mindset and other irrational beliefs influence the psychological well-being of the athlete.
5	The mediation effect of Perceived Social Support and Perceived Stress on the relationship between Emotional Intelligence and Psychological Wellbeing in Male Athletes	Romualdas Malinauskas, Vilija Malinauskiene	Journal of Human Kinetics	2018	398 male athletes	- **Schutte Self-Report Inventory**- (Schutte et al., 1998). - **Multidimensional Scale of Perceived Social Support** (MSPSS; Zimet et al., 1988). - **Perceived Stress Scale–10** (PSS-10) (Cohen et al., 1983). -**Ryff psychological wellbeing scale** -RPWBS- (Ryff, 1989).	Results from structural equation modelling procedures identified that perceived social support and perceived stress partially mediated the association between emotional intelligence (EI) and psychological well-being. The sequential mediation effects of perceived social support–perceived stress on the relations between EI and well-being.	Perceived social support and perceived stress partially mediated the association between emotional intelligence and psychological wellbeing.
6	Mental toughness, optimism, pessimism, and coping among athletes	Adam R. Nicholls, Remco C.J. Polman, Andrew R. Levy, Susan H. Backhouse	Personality and Individual Differences	2008	677 athletes (male 454; female 223) aged between 15 and 58 years.	**- Coping inventory for competitive sport** (CICS; Gaudreau & Blondin, 2002) **- Mental Toughness Questionnaire** (MTQ48; Clough et al., 2002). **- Life Orientation Test** (LOT; Scheier & Carver, 1985)	Results show that mental toughness correlated significantly with 8 of the 10 coping subscales and optimism. In particular, higher levels of mental toughness were associated with more problem or approach coping strategies (mental imagery, effort expenditure, thought control, and logical analysis) but less use of avoidance coping strategies (distancing, mental distraction, and resignation). Eight coping subscales were significantly correlated with optimism and pessimism.	Mental toughness correlated significantly with 8 of the 10 coping subscales and optimism. Must to include coping and optimism training in mental toughness interventions.
7	Distinguishing psychological characteristics of expert cricket batsmen	Weissensteiner, Juanita R.;Abernethy, Bruce; Farrow, Damian; Gross, John	Journal of Science and Medicine in Sport	2012	Adult-aged batsmen of two different skill levels (highly skilled *n* = 11; less skilled *n* = 10)	-**Mental Toughness Inventory**. -**Multidimensional Perfectionism Scale**, MPS. -**Athletic Coping Skills Inventory-28**, ACSI-28. -**Attributional Styles Questionnaire**, ASQ.	The results show the descriptive data for each of the skill groups for the respective total and subscale scores of each inventory. Univariate analyses of the overall scores for the MTI, MPS, ACSI-28 and ASQ revealed that the highly skilled batsmen scored significantly higher than the older, more experienced, less skilled batsmen for the Overall Mental Toughness Score (F (1, 19). Overall scores for the MPS, ACSI-28 and ASQ were not found to be significant discriminators	Predicting mental toughness at an early age can be useful in detecting and developing talents
8	Relationship between optimism/dispositional pessimism, performance and age in competitive soccer players.	Garcia Naveira, Alejo; Diaz Morales, Juan Francisco	Revista Iberoamericana de Psicología del ejercicio y el deporte.	2010	151 male soccer players aged between 14 and 24 years old	**Life Orientation Test** (LOT-R; Scheier, Carver y Bridges,1994).	Optimism is presented as an important variable to study in understanding the psychological characteristics associated with maximum performance. The results indicate a positive and moderate relationship between optimism and sports performance. Furthermore, a non-significant trend is obtained in the optimism trait depending on the age category, with optimism increasing as the age of the athletes increases.	The results indicate a moderate and positive relationship between optimism and athletic performance.
9	Optimism as a contribution to sports resilience.	Reche, Cristina; Gomez-Diaz, Magdalena; Martinez-Rodriguez, Alejandro; Tutte, Veronica	Revista Iberoamericana de Psicología del ejercicio y el deporte.	2018	87 women and men fencing athletes (Ages M = 25.2; DT = 10.6).	**-Resilience Scale** (Wagnild and Young, 1993). **-Life Orientation Test** (LOT-R; Scheier, Carver y Bridges,1994).	The results show that 17% of the participants present a high level of resilience. 85.1% are optimists as opposed to 14.9% that are not (pessimist). No differences of resilience or optimism were found by gender or sports level, but there were differences found among the analysed age groups the subjects belong to, favouring the seniors (older than 18 years of age) where resilience was most present. Resilience and optimism are related.	There were differences found among the analyzed age groups the subjects belong to, favoring the seniors (older than 18 years of age) where resilience was most present. Resilience and optimism are related.
10	Coping, optimism and satisfaction with life among Spanish and Polish football players: a preliminary study.	Canton Chirivella, Enrique; Checa, Irene; Budzynska, Natalia	Revista de Psicología del deporte	2013	61 football players from Poland and Spain.	**- Life Orientation Test** (LOT-R; Scheier, Carver y Bridges,1994). **-The Life Satisfaction Scale** (Diener, Emmons, Larsen, and Griffin 198) -**The Coping Inventory for Stressful Situations** -CISS- (Endler and Parker, 1990)	The Spanish players achieved significantly higher scores than the Poles in the use of emotion-related coping strategies (*p* = .001), with the obtainment of a relevant effect size (> 0.130) and high power (0.942). In addition, the Polish players were found to be significantly more optimistic than the Spanish (*p* = .025).	There are cultural differences when it comes to using coping strategies.
11	Perfectionistic strivings, perfectionistic concerns, and reactions to poor personal performances among intercollegiate athletes	Lizmore, Michael R.;Dunn, John G. H.;Causgrove Dunn, Janice	Psychology of Sport and Exercise	2017	239 athletes (140 men, 99 women) (M age = 20.50 years, SD = 1.99)	-**Sport-Multidimensional Perfectionism Scale-2** (Sport-MPS-2: Gotwals & Dunn, 2009) **-Multidimensional Inventory of Perfectionism in Sport** (MIPS: Stoeber, Otto, & Stoll, 2006). **-Short Form of the Self-Compassion Scale** (SCS-SF: Raes, Pommier, Neff, & Van Gucht, 2011) **-Life Orientation Test** (LOT: Scheier & Carver, 1985) **-Rumination about an Interpersonal Offense Scale** (RIO: Wade et al., 2008)	Standardized regression coefficients from hierarchical regression analyses indicated that perfectionistic concerns were negatively associated with self-compassion and optimism, and positively associated with pessimism and rumination (all *p*s < 0.001), whereas perfectionistic strivings were positively associated with self-compassion and optimism, and negatively associated with pessimism (all *p*s < 0.01).	There are important links between perfectionistic strivings, perfectionistic concerns, and athletes’ cognitive reactions to personal failure in competitive sport.

## Discussion

The usual position of the researcher of psychological variables that affect sports performance is to look for relationships between ‘negative’ variables, first in the form of basic psychological processes, or distorting cognitive behavioural, unpleasant or evaluable as deficiencies or problems, in a psychology for the ‘risk’ society, which emphasises the rehabilitation that stems from overcoming personal and social pathologies [31], and, lately, regarding the affectation of the athlete’s mental health [32]. This fact seems to be true in many cases and situations and to openly contradict the proclaimed psychological benefits of practising sports (among others: Cantón [33], ; Froment and González [34]; Jürgens [35]).

However, it is possible to adopt another approach focused on the ‘positive’ variables, also in relation to the athlete’s performance. This has been the main objective of this systematic review of the existing literature and far from being a novel approach, although a minority one, it fits perfectly with the definition of our area of knowledge in the broad field of health, as has been pointed out for some time [36, 37].

After carrying out the aforementioned systematic review, a relatively low number of articles were identified by experts that met the established conditions—according to the PRISMA method [37–40]—regarding databases, keywords, and exclusion and inclusion criteria. These precautions were taken to obtain the most accurate results possible, and thus guarantee the quality of the conclusions.

The first clear result that stands out is the great difficulty in finding articles in which sports ‘performance’ is treated as a well-defined study variable adapted to the situation and the athletes studied. In fact, among the results (11 papers), only 3 associate one or several positive psychological variables with performance (which is evaluated in very different ways, combining objective measures with other subjective ones). This result is not surprising, since in several previous studies (e.g. Nuñez et al. [41]) using a systematic review, this relationship is found to be very weak and nuanced by the role of different mediating factors, such as previous sports experience or the competitive level (e.g. Rascado, et al. [42]; Reche, Cepero & Rojas [43]), despite the belief—even among professional and academic circles—that there is a strong relationship between negative variables and poor performance, and vice versa, with respect to the positive variables.

Regarding what has been evidenced in relation to the latter, even with these restrictions in the inclusion and exclusion criteria, and the filters applied to the first findings, a true ‘galaxy’ of variables is obtained, which also belong to different categories and levels of psychological complexity.

A preliminary consideration regarding the current paradigm of sport psychology: although it is true that some recent works have already announced the swing of the pendulum on the objects of study of PD, by returning to the study of traits and dispositions, and even to the personality of athletes [43–46], our results fully corroborate this trend. Faced with five variables present in the studies selected at the end of the systematic review, a total of three traits/dispositions were found, which were also the most repeated—optimism being present in four articles, mental toughness present in three, and finally, perfectionism—as the representative concepts of this field of psychology, which lately, as has already been indicated, is significantly represented in the field of research in this area [46–52]. In short, the psychological variables that finally appear in the selected articles are: psychological well-being (PWB) [53]; self-compassion, which has recently been gaining much relevance with respect to the positive attributional resolution of personal behaviours [54], satisfaction with life (balance between sports practice, its results, and life and personal fulfilment [55], the existence of approach-achievement goals [56], and perceived social support [57]). This last concept is maintained transversally in several theoretical frameworks, such as Sports Commitment [58].

The most relevant concept, both quantitatively and qualitatively, supported by the fact that it is found in combination with different variables and situations, is not a basic psychological process, but a high-level cognitive construct: psychological well-being, in its eudaimonic aspect, first defined in the general population by Carol Ryff [59, 60] and introduced at the beginning of this century in sport (e.g., Romero, Brustad & García-Mas [13], ; Romero, García-Mas & Brustad [61]). It is important to note that this concept understands psychological well-being as multifactorial, including autonomy, control of the environment in which the activity takes place, social relationships, etc.), meaning personal fulfilment through a determined activity and the achievement or progress towards goals and one’s own objectives, without having any direct relationship with simpler concepts, such as vitality or fun. In the selected studies, PWB appears in five of them, and is related to several of the other variables/traits.

The most relevant result regarding this variable is its link with motivational aspects, as a central axis that relates to different concepts, hence its connection to sports performance, as a goal of constant improvement that requires resistance, perseverance, management of errors and great confidence in the possibility that achievements can be attained, that is, associated with ideas of optimism, which is reflected in expectations of effectiveness.

If we detail the relationships more specifically, we can first review this relationship with the ‘way of being’, understood as personality traits or behavioural tendencies, depending on whether more or less emphasis is placed on their possibilities for change and learning. In these cases, well-being derives from satisfaction with progress towards the desired goal, for which resistance (mental toughness) and confidence (optimism) are needed. When, in addition, the search for improvement is constant and aiming for excellence, its relationship with perfectionism is clear, although it is a factor that should be explored further due to its potential negative effect, at least in the long term.

The relationship between well-being and satisfaction with life is almost tautological, in the precise sense that what produces well-being is the perception of a relationship or positive balance between effort (or the perception of control, if we use stricter terminology) and the results thereof (or the effectiveness of such control). This direct link is especially important when assessing achievement in personally relevant activities, which, in the case of the subjects evaluated in the papers, specifically concern athletes of a certain level of performance, which makes it a more valuable objective than would surely be found in the general population. And precisely because of this effect of the value of performance for athletes of a certain level, it also allows us to understand how well-being is linked to self-compassion, since as a psychological concept it is very close to that of self-esteem, but with a lower ‘demand’ or a greater ‘generosity’, when we encounter failures, mistakes or even defeats along the way, which offers us greater protection from the risk of abandonment and therefore reinforces persistence, a key element for any successful sports career [62].

It also has a very direct relationship with approach-achievement goals, since precisely one of the central aspects characterising this eudaimonic well-being and differentiating it from hedonic well-being is specifically its relationship with self-determined and persistent progress towards goals or achievements with incentive value for the person, as is sports performance evidently [63].

Finally, it is interesting to see how we can also find a facet or link relating to the aspects that are more closely-related to the need for human affiliation, with feeling part of a group or human collective, where we can recognise others and recognise ourselves in the achievements obtained and the social reinforcement of those themselves, as indicated by their relationship with perceived social support. This construct is very labile, in fact it is common to find results in which the pressure of social support is hardly differentiated, for example, from the parents of athletes and/or their coaches [64]. However, its relevance within this set of psychological variables and traits is proof of its possible conceptual validity.

## Conclusions

Analysing the results obtained, the first conclusion is that in no case is an integrated model based solely on ‘positive’ variables or traits obtained, since some ‘negative’ ones appear (anxiety, stress, irrational thoughts), affecting the former.

The second conclusion is that among the positive elements the variable coping strategies (their use, or the perception of their effectiveness) and the traits of optimism, perfectionism and self-compassion prevail, since mental strength or psychological well-being (which also appear as important, but with a more complex nature) are seen to be participated in by the aforementioned traits.

Finally, it must be taken into account that the generation of positive elements, such as resilience, or the learning of coping strategies, are directly affected by the educational style received, or by the culture in which the athlete is immersed. Thus, the applied potential of these findings is great, but it must be calibrated according to the educational and/or cultural features of the specific setting.

### Limitations

The limitations of this study are those evident and common in SR methodology using the PRISMA system, since the selection of keywords (and their logical connections used in the search), the databases, and the inclusion/exclusion criteria bias the work in its entirety and, therefore, constrain the generalisation of the results obtained.

Likewise, the conclusions must—based on the above and the results obtained—be made with the greatest concreteness and simplicity possible. Although we have tried to reduce these limitations as much as possible through the use of experts in the first steps of the method, they remain and must be considered in terms of the use of the results.

### Future developments

Undoubtedly, progress is needed in research to more precisely elucidate the role of well-being, as it has been proposed here, from a bidirectional perspective: as a motivational element to push towards improvement and the achievement of goals, and as a product or effect of the self-determined and competent behaviour of the person, in relation to different factors, such as that indicated here of ‘perfectionism’ or the potential interference of material and social rewards, which are linked to sports performance—in our case—and that could act as a risk factor so that our achievements, far from being a source of well-being and satisfaction, become an insatiable demand in the search to obtain more and more frequent rewards.

From a practical point of view, an empirical investigation should be conducted to see if these relationships hold from a statistical point of view, either in the classical (correlational) or in the probabilistic (Bayesian Networks) plane.

The results obtained in this study, exclusively researched from the desk, force the authors to develop subsequent empirical and/or experimental studies in two senses: (1) what interrelationships exist between the so called ‘positive’ and ‘negative’ psychological variables and traits in sport, and in what sense are each of them produced; and, (2) from a global, motivational point of view, can currently accepted theoretical frameworks, such as SDT, easily accommodate this duality, which is becoming increasingly evident in applied work?

Finally, these studies should lead to proposals applied to the two fields that have appeared to be relevant: educational and cultural.

### Application/transfer of results

A clear application of these results is aimed at guiding the training of sports and physical exercise practitioners, directing it towards strategies for assessing achievements, improvements and failure management, which keep them in line with well-being enhancement, eudaimonic, intrinsic and self-determined, which enhances the quality of their learning and their results and also favours personal health and social relationships.

## Data Availability

There are no further external data.
